# ‘I take the drugs… to make the sickness to move out of me’: key populations’ and service provider perspectives about facilitators and barriers to ART adherence and retention in care in Nigeria

**DOI:** 10.1186/s13690-024-01282-9

**Published:** 2024-06-17

**Authors:** Osasuyi Dirisu, George I. E. Eluwa, Steve Callens, Eseoghene Adams, Akinola Akinwunmi, Scott Geibel, Isa Iyortim

**Affiliations:** 1 Policy Innovation Center, Snr Fellow Nigerian Economic Summit Group, Abuja, Nigeria; 2 Diadem Consults Initiative, Abuja, Nigeria; 3 Faculty of Medicine and Health Sciences, University of Gent, Ghent, Belgium; 4 Population Council, Washington, DC USA; 5 Policy Innovation Center, Abuja, Nigeria; 6 United States Agency for International Development, Abuja, Nigeria

**Keywords:** Adherence, Antiretroviral therapy, Retention, Key population, Facilitators, Barriers, Nigeria

## Abstract

**Background:**

Adherence to antiretroviral therapy (ART) has individual and public health benefits and is critical to improving life expectancy, achieving viral suppression, and reducing the risk of HIV transmission. This qualitative study explored the experience of receiving care as well as perceived facilitators and barriers of treatment initiation, retention in ART care, and adherence to treatment.

**Methods:**

In-depth interviews were conducted among 28 men who have sex with men (MSM) and female sex workers (FSWs) receiving ART services in Lagos and Benue states. Key informant interviews were also conducted among 16 service providers engaged in counselling, clinical care, and ART treatment for MSM and FSWs. The Social Ecology Model guided the exploration of perceived barriers and facilitators of treatment initiation, retention in ART care and adherence to treatment. Qualitative data analysis was managed using NVIVO 11 software and themes were analysed using thematic analysis.

**Results:**

We found that the key barriers to ART adherence were low motivation to comply with medication regimen, work commitments, socioeconomic factors, stigma, negative provider attitude and distance to health facilities. Facilitators of adherence identified include the desire to live a productive life, strong family support and participation in support group programs. Comprehensive adherence counselling, support group programs and an effective follow-up system were factors identified by service providers as key to facilitating adherence.

**Conclusion:**

To be effective, ART programs must address the unique challenges key populations face in accessing treatment and achieving optimal adherence regarding establishing a strong support system and follow-up. Community level interventions that support a stigma-free environment are critical to sustaining engagement in care.

**Table:** 

**Text box 1. Contributions to the literature**
• Key populations have a higher burden of HIV and thus programs that can optimize adherence to treatment is vital to mitigate the cycle of HIV transmission within key populations and between key populations and the general population.
• Adherence to live long medication remains a complex phenomenon that is influenced by diverse factors at the individual, environmental and institutional levels.
• Public health programs that provide comprehensive HIV care and treatment among key populations must be robust and adaptive to accommodate and mitigate the barriers to optimal adherence to treatment.

## Background

The prevalence of HIV in Nigeria in 2018 was 1.4% and it is estimated that 1.9 million people are HIV-infected [1]. Sub Saharan Africa (SSA) region is most affected as it contributes about 70% of all HIV infections with Nigeria accounting for more than half of new HIV infections and deaths in the region [2]. HIV prevalence among key populations (KPs) is significantly higher than that of the general population and they contribute to 62% of new infection [2–5]. In Nigeria, HIV prevalence among female sex workers (FSWs) and men who have sex with men (MSM) is 14.4% and 23%, respectively [2]. Despite their greater vulnerability to HIV, there are gaps in access to prevention and treatment services for KPs due to the restrictive policy and social environment [6] such as the same-sex marriage prohibition law established in 2014 (SSMP). The wide sweeping SSMP law asides prohibiting marriage between same sex, proposes a 10 – 14 year jail term for cohabitation between same-sex partners, the registration, operation, or participation in gay clubs, societies or organization as well as those who support such activities [7].

Uptake and adherence to anti-retroviral treatment (ART) is critical in the management of HIV/AIDS and reduction of HIV associated morbidity and mortality [8–11]. Optimal adherence is even more critical for hidden and underserved populations and is beneficial to public health^10^. Early initiation of ART and high levels of adherence at least 95% is required for optimal viral suppression and improvement in life expectancy [9, 12, 13]. Twenty-three percent of Africans on ART achieve less than 80% level of adherence [14]. A national survey in Nigeria showed that among those who knew their HIV status and self-reported being on ART or had detectable antiretrovirals via laboratory assay, 81% of them were found to be virally suppressed and this was higher among women (82% compared to men 79%) [15]. Effective ART adherence lowers the risk of HIV transmission, helps achieve viral suppression, and improves quality of life [8–10, 16, 17]. Conversely, poor adherence has been associated with antiretroviral drug resistance treatment failure, virological failure, declining CD4 counts, opportunistic infections, and poorer health outcomes [10, 13, 17, 18]. The success of an ART program is hinged on adherence to the medication regimen [8, 11].

Two key challenges experienced by ART programmes include poor adherence to treatment and defaulting from treatment [18]. Monitoring ART adherence and follow up of patients can be done by patient self-report, pill counts, pharmacy refill records, drug level monitoring and physician assessment [11]. Some factors which influence adherence to ART include patient’s age, regimen complexity, drug side-effects, advanced HIV disease and patient’s mental health [17]. Other factors include forgetfulness, busy schedule, treatment burden and side effects [16, 18–20]. Barriers such as financial constraints (cost of drugs and transport, distance to access treatment) as well as fear of disclosure and stigmatization are common in developing countries [18, 21, 22]. Criminalization of same-sex activity in Nigeria as well as stigma and discrimination experienced by FSW and MSM affect the uptake of HIV services in Nigeria [23]. Institutional barriers to ART adherence at health facilities include long waiting lines and negative experiences with clinical staff [16, 19]. Facilitators of ART adherence includes personal commitment to adhere, professional support, noticeable health improvements after the start of ART, perceived need to meet up with family responsibilities, support received from others and adherence counselling [17, 19].

Although these facilitators and barriers have been well documented for general population, little is known about the perceived barriers and facilitators for ART adherence among KPs particularly MSM and FSWs in Nigeria. In addition, developing focused strategies that improve ART adherence require an understanding of gaps that can be addressed in HIV treatment programs for KPs. It is therefore important to qualitatively explore the perceived facilitators and barriers to accessing ART from the provider and client perspective. This study used the socio-ecological model to explore factors that facilitate or hinder ART adherence among KPs in Lagos and Benue State, Nigeria.

## Methodology

### Study design

This qualitative study was conducted in November 2018 as part of a nested study that assessed Test and Start (TnS) service delivery models for MSM and FSWs in two states (Lagos and Benue) in Nigeria. Six hundred and five participants who tested positive and enrolled in treatment were recruited into the study and followed up for twelve months to measure ART retention and behavioural outcomes of interest. This study was conducted among a purposive sample of the recruited participants to explore the experience of receiving care as well as perceived facilitators and barriers of treatment initiation, retention in ART care and adherence to treatment. In accordance with the purposive sampling methodology [24], we identified specific characteristics needed and participants were chosen on purpose using the following criteria; being HIV positive and having had at least one drug pick up since treatment initiation.

### Study setting

This study was conducted in Lagos in South West Nigeria and Benue in North Central Nigeria. The 2016 projected population of Lagos and Benue states was 12.6 and 5.7 million people, respectively, according to the National Bureau of Statistics. Lagos state is the smallest state by geographic size but has 27.4% of the urban population of Nigeria [25]. Lagos is one of the most ethnically diverse states in Nigeria as a result of increasing rural to urban migration compared to Benue that is the 11th largest state by geographic size, less diverse and largely peri-urban to rural [26].

In Lagos, the three study sites used provide a full range of HIV services supported by one partner only. In Benue, the two study sites used were drop-in centres where KP services and ART services are provided by different partners. HIV prevalence among MSM in 2014 was 41% in Lagos state and 37% in Benue state. The prevalence of HIV among brothel and non-brothel based FSW in Benue was 14% and about 8% for both brothel and non-brothel based FSW in Lagos state in 2019 [1, 2].

### Study participants and data collection procedure

In-depth interviews (IDIs) were conducted among 28 study participants (14 MSM and 14 FSW) in both states who tested positive, had initiated ART treatment and had at least one drug pickup visit after commencing treatment. Clients were asked during follow-up visits if they were interested in participating in an interview; if they agreed, written informed consent was obtained and the IDIs were conducted in a private room by trained interviewers. Interviews were conducted by trained qualitative researchers with extensive experience in qualitative data collection and working with key populations.

They were also trained on the study tools and research ethics. All interviews were conducted in English.

Key informant interviews (KIIs) were also conducted among 16 KP service providers in both states who were engaged in some aspect of clinical services such as counselling, clinical care and consultation, ART treatment, pharmacy and laboratory services at the facilities for at least three months. The KIIs were conducted to understand the challenges providers face in delivering care and the kind of support they need to improve adherence to treatment. The facility officers in charge were contacted to provide information about the service providers who provide the services of interest. They were then invited to participate in the KIIs, if the providers agreed. The interviewer scheduled a time convenient to the service provider.

The Social Ecology Model (SEM) guided the exploration of perceived barriers and facilitators of treatment retention in ART care and adherence to treatment. Semi-structured IDI and KII guides were developed to stimulate discussions about barriers and facilitators of care at individual, interpersonal, community and institutional levels.

### Data analysis

The interviews were recorded digitally, transcribed verbatim, and transferred to NVivo 11 software for analysis. The codebook development process entailed a review of all the transcripts by two researchers who contributed to the development of a thematic framework of codes through consensus. Thematic analysis was used as an analytical strategy to explore patterns and themes within the data. The process of identifying themes highlighted contextual situations that underpin perceptions and experiences expressed in the qualitative data and the themes were organized using the SEM.

### Ethical considerations

The study protocol received ethical approvals from Population Council’s Institutional Review Board, U.S.A and the National Health Research Ethics Committee (NHREC), Nigeria. The procedures involving human participants complied with Population Council’s Institutional Review Board and the NHREC ethical standards for the conduct of research. Written informed consent was obtained from all participants by trained interviewers prior to commencement of the interviews.

## Results

Fourteen MSM and fourteen FSWs participated in the IDIs across the two states. Over half of the KPs were aged 25-34 years and the majority were single (Table 1). The service providers included adherence counsellors, pharmacists, doctors, nurses and laboratory technicians; majority were aged 25-34 years old.

**Table 1:** Demographic characteristics of MSM, FSW, and service provider participants

**Description**		**Number of interviewees (*N* = 44)**
**Service Providers**		**16**
**KPs (MSM and FSW)**		**28 (14 MSM and 14 FSWs)**
**Demographic features for providers**		**Number of Providers (*N* = 16)**
**Age Group**	25-34	12
	35 and Above	4
**Marital Status**	Single	7
	Married	7
	Other (widowed/separated)	2
**Categories**	Adherence Counsellor	3
	Lab technician	6
	Doctor	3
	Nurse	2
	Pharmacist	2
**Demographic features for KP**		**Number of KPs (*N* = 28)**
**Age Group**	16-24	6
	25-34	16
	35 and Above	6
**Marital status**	Single	21
	Married	3
	Other (widowed/separated)	4
**Categories (KP)**	MSM	14
	FSW	14

**Fig. 1:**
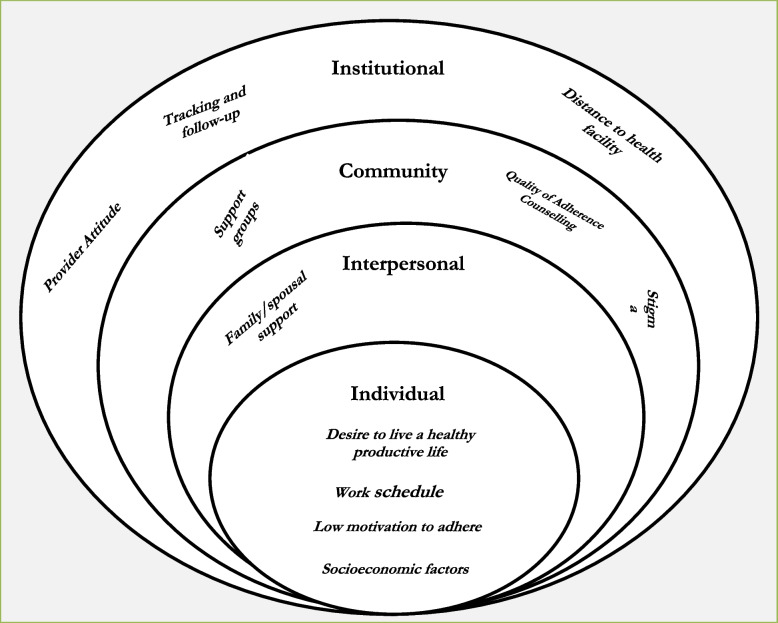
Socioecological model on client and provider perspectives about HIV care and adherence

Findings are discussed along four thematic areas: individual factors, interpersonal factors, community factors and institutional factors. A summary of client and provider perspectives about care and adherence are presented in Fig. 1.

### Clients’ perception about care and adherence

#### Individual/client-level factors

##### Desire to live a healthy productive life

The desire to live a healthy life was considered a major motivating factor for commencing ART. Some participants reported that they were inclined to adhere to ART because they were counselled to understand that it was possible to live productive lives and contribute meaningfully to society after HIV diagnosis.

‘I don’t want the sickness… because I don’t want it in my life! I don’t want it in my body! You understand what I’m saying? I take the drugs so that it would make the sickness to move out of me, you understand what

“I’m saying? So, that is why it is not… difficult for me. Anytime they give me appointment, I will come. You understand?” (MSM_Lagos).

“I know I am positive. So, what use will it be for me when I know this drug is going to keep me longer? Why should I want it to miss or go against what I shouldn’t do? This keeps me going. Why should I want it to leave it? This is like my life – if I stop, I know the consequences to stop. So, why should I want to mess with it? I was told I shouldn’t miss it twice a month – so, why should I want to jeopardize with my life?” (MSM_Lagos).

Although some were initially reluctant to commence treatment, counselling played a vital role in facilitating the decision to commence ART. They understood that adherence was critical to improving their health and wellbeing.

“I was in shock, and refused treatment for over a year, when I was knocked down by fever… I said, let me take this bull by the horn and live this life” (MSM_Benue).

“The person who tested me, explained that even though I have the virus, it is not the end of the world, there are many people out there with the virus, they are still living sound provided I take my drugs… He also explained to me that the fact that I am positive does not mean that I don’t have something to offer to the community and I should adhere to my drugs” (FSW_Benue).

##### Work schedule

Some participants reported that they found it difficult to make out time to attend the clinics on their ART appointment days due to work commitments or work-related travel. To meet up with their ART appointment days, they had to take permission from their employers, and they were reluctant to do so because of the fear of being stigmatized if they had to disclose the reason for their frequent clinic visits.

“Today I took … permission I didn’t tell them I’m HIV I just told them I’m beginning to see changes in my body I needed to go see a... I’ve been coming here for re-fill, I come here when I’m off or its holiday for me. Like the last time I came here… we had a day off so that’s was why I was able to come get a re-fill” (MSM_Lagos).

##### Low motivation to adhere

Some participants admitted that poor motivation to adhere to the medication regimen was a barrier to adherence. The dietary changes that were required and the cumbersome nature of the instructions were also considered reasons for the low motivation to adhere. The use of reminders such as alarms were recommended by service providers to help clients remember to take their medication at the right time.

“I was tired of it. I was… like; what is this? Sometimes I will forget… I was being advised to start using alarm – I set the alarm. Sometimes the laws behind the taking of the drug is too much: two hours before, two hours after; don’t eat oily food…the thing was just new to me so at a time, I just stopped. I won’t lie. I started missing my appointment, when my drugs get finished, I wouldn’t go to get a refill. To the extent that there was a day I got there, a lady was very angry with me and was like ‘do I want to kill myself?’… ever since then I talked to myself that ‘okay, it’s for my own good’ and since then, I’ve started taking” (FSW_Lagos).

“I procrastinate a lot so it’s more like I will do it, it’s just 9 o’clock, funny enough I know when it’s 9 o’clock, so I don’t need an alarm to tell me this is 9 o’clock but sometimes I be like I will take it, after all what will a few minutes do. Few minutes might now turn to 30 minutes, 30 minutes will turn to an hour and busy doing some other thing just busy procrastinating, holding off…” (MSM_Benue).

##### Socioeconomic factors

Socio economic factors were considered barriers to consistently keeping up with clinic appointments. Some interviewees reported that appointments were missed due to lack of transport fare and having adequate food to eat was considered a priority. In addition, the opportunity cost of losing income if they left their businesses unattended to visit the clinics for medication pickup was a consideration. The provision of transport support by the program was considered a vital incentive to keeping up with appointments.

“Yes, (poverty) is part of it, number 1. But some other people they would be thinking it that way, some people might even come to clinic that day, they might not have eaten, they would be saying, ah! They asked me to come and collect drugs again, whereas I’ve not even sold anything at the market, oh God! … but once you give them the transport money it would make them to say they know they have received something” (FSW_Lagos).

### Interpersonal factors

#### HIV disclosure and family/spousal support

Family/spousal support was identified as one of the most important facilitators of adherence. Some participants reported that the support they received from close family members in the form of reminders encouraging them to take their drugs, exempting them from strenuous domestic tasks and helping them pick up refills was invaluable in facilitating medication adherence. In addition to family support, some participants received support from friends to continue medication.

“They (my family) really supported me… I am now 25 years old, when I discovered this, I was 20 years old and I was just getting into school. So, they really supported me in the sense that at times, they come get my drugs for me because I didn’t school in Lagos; I schooled in Owerri. At times, they come help me get my drugs, they waybill (send it by courier) it to me and at times when I don’t have that transport to come, they’re always on my side…” (MSM_Lagos).

“My children also use to encourage me anytime… even the little ones. When I gave birth, he will say mummy… take your drugs o!. You know I will work… and get tired so when I go to bed, they will say mummy take your drugs o, you haven’t taken your drugs, so they are very encouraging. Like my husband, if I didn’t come to hospital because sometimes, they give me for two months, he will remind me to go to the hospital again… I will now tell him they gave me for two months” (FSW_Benue).

“Not all my friends know about my status, but some know. At times like my friends, they will say bros, you don’t take your drugs? I will say, hmm I forget, I don too tire, they will be like, take it now. So, I believe they are a very good impact to me” (MSM_Benue)

In some instances, participants noted that it was unwise to disclose their HIV status to friends and family because the reaction to their HIV status and associated stigma may hinder uptake of treatment.

“No! Nobody…the only people who knows about my HIV status are the people here (in the ART clinic) … I don’t talk to anybody about it. The last person I was in sexual relationship… this person does not really know. I’m sure I got this from this person because I know that every time, I had sex, there’s usually changes in my body I feel like there’s something inside me” (MSM_Lagos).

### Community factors

#### Support groups

Support group meetings provided a friendly atmosphere for most participants to listen to success stories and learn from their peers about how to manage challenges they encounter in managing medication pickup and use. A wide range of health education topics such as nutrition, health lifestyle, STI prevention were taught during these meetings. The support groups were also key to providing emotional and financial support for members, facilitating follow-up of members, and clarifying misconceptions about HIV diagnosis and treatment. Service providers also echoed the value of support group meetings as avenues to deliver health talks and encourage clients on the importance of ART adherence.

“The name of our meeting here is “Friends of Integrity” so I will call it friends and daughters. There are some friends around my place that I even brought them down here so anytime we met we used to encourage ourselves how we take our drugs… Some of them that are not physically fit and their finance is not well and suffer before they get something to eat… when they come to me anything I have I will give them so that it will help them to eat so that they will take their drugs. So, we use to help ourselves in advice and anything we have we share between ourselves” (FSW_Benue)

“ I am always talking about the support group meeting because it was there, we got so many things. When we started the support group meeting, it was there that they told us things to do, and things we are not supposed to do, the kind of things we are supposed to eat and those that are not good for us… toxic chemicals that kills... the effect of the drugs on you, so even if you are taking the drugs but you are taking some certain things, it won’t work. It was basically on our nutrition so if you avoid this, you stay healthy but if you are going on this, you should know that even taking drugs, you won’t be as healthy as you are supposed to” (FSW_Benue).

“We set up this support group meetings in order to encourage the clients on the importance of taking their drugs regularly which has actually proven a success to the programme. They come immediately you give them a talk about ART, not missing their drugs in that same meeting we give them some health talks, they come regularly picking up their drugs as usual” (SERVICE PROVIDER_Benue).

#### Stigma

The fear of being stigmatized in the process of receiving care or using ART was considered a major barrier to adherence. Some participants reported that societal perception about HIV/AIDS affected the way they were viewed by people who knew their status. Others reported they missed their medication if they were in situations where they felt they could be judged if they were seen using medication.

“Sometimes If you are in a place where people are… if you have your drugs there, you will be afraid of bringing it out… I will not be able to bring it out until I leave that place. But by that time, the time has passed” (FSW_Lagos)

“Yes, I’ve been stigmatized before. A guy that claimed he had issues with me… even though I never had any issues with him, he posted online that I died even when I was still alive… that I died that morning with HIV. And it was publicized. I felt bad. For like 3 weeks; I wasn’t myself. I couldn’t come out, I couldn’t talk to people, I was seeing myself like a ghost. People were seeing me like a ghost. When somebody came in and he was like; “I just want to be sure you’re fine. I heard the news”. Somebody stigmatizing me even when I’m alive” (MSM_Lagos)

### Institutional factors

#### Provider attitude

There was consensus that the negative attitude of service providers towards people living with HIV discouraged participants from visiting the facilities for follow-up appointments or taking medication. Some participants reported that service providers spoke condescendingly to them when they presented at the health facilities for treatment and follow-up because of their HIV status. Service providers were sometimes reported to go beyond the limits of the professional relationship to ask sensitive questions or make comments that made participants uncomfortable. Overall, poor provider-client relationship was considered a barrier to adherence.

“When you come to a hospital and especially the health provider make you feel… it’s because you’re into this (HIV) that’s why they’re talking to you like that. There is a lot of low self-esteem I mean; you feel like it’s because you’re like this and they’re not like this that’s why they’re talking to you like this... As a human being you start thinking many things. Sincerely speaking,… it can actually stop you from taking your drugs**”** (MSM_Benue).

“That particular girl (service provider)! The way she does things, I don’t like them. You understand? She asks some questions that are not in the book that… she is not supposed to ask some questions. So, it’s not all the questions that she asks that I answer… sometimes she will take things as a playful thing while me myself, I am the one that is suffering the sickness and it’s paining me. You understand?” (MSM_Lagos)

#### Distance to health facility

Distance to health facility and transportation cost was viewed as a critical factor in meeting up with appointments. Clients who visit the health facility for drug pick-up were reimbursed for transportation costs by the ART program. Although the reimbursement received to cover transportation costs was considered very encouraging, some participants opined that it was challenging to travel far for medication pickup. Participants sometimes missed appointments if they didn’t have transport money because the cost of transportation was reimbursed after the clinic visit.

“My location, the proximity. At times I don’t have money to come on that day I am having my appointment, so at times, maybe I will skip, then the next appointment when I have money, then I will come” (MSM_Benue)

“in this facility, we don’t talk about money, the first time you do come they pay for your fare… they give you money for transport, and we come for support group meeting, they still pay our transport… we have refreshment, we’ve never been asked for one kobo… when you have STI, you are being treated for free, you will be given drugs… so you can’t tell me that err, poverty will stop you from collecting the drugs no, in other facilities they might be doing it, but here, they can, they even support us” (FSW_Benue).

### Providers’ perspectives about care and adherence

#### Adherence counselling

Service providers emphasized the importance of comprehensive adherence counselling in helping clients settle into treatment and improve decision making regarding adherence. Laboratory personnel described client expectation on viral load suppression as a motivating factor for adherence. Clients were always eager to see that their results were improving as an indication that they were making progress with treatment.

“Our adherence counselling is robust, we have adherence counselling at different levels of the service delivery, at every service delivery point. So, starting from the field, that is when they are reached, and they first know their status to when they are linked to…. the clinic. So, from the adherence counsellor’s table to the lab scientist for the testing, there is adherence counselling… at the nurse or pharmacist point there is adherence… at about five service delivery points. And every person, every member of the clinic staff you meet delivers adherence messaging”. (SERVICE PROVIDER_Benue).

“Adherence has been better because we have intensified our adherence session, so I think we tried to let them know what they are going into. So, we don’t do the rush, you are not ready; we allow you to go home and think about it and come back. Fine, its test and start, if I test you and you start and you’re not ready to continue, it’s not successful, so we allow them to digest it, accept it before we give them, and it has actually helped our adherence to do well too” (SERVICE PROVIDER_Lagos).

Adherence counselling was considered more comprehensive when it was provided at the facility compared to being provided at the community. Counselling at the facility was considered more comprehensive than at the community because community personnel were under pressure to attend to the large number of clients who show up for HIV testing and meet their daily testing target. In addition, adherence counsellors were not always available at the community and clients may have to wait for extended periods to receive counselling which may be less thoroughly done compared to the clinic settings. This may also have affected the quality of adherence counselling sessions during community outreaches.

“You know when you go to the outreach… you are given target. So the time for adherence counselling is not much; everybody, they’re rushing – one; I want to meet to meet my target; two – I want to test the other clients especially when you see about three – four – five people standing before you; want to get them tested. So, facility is the best place to give an adherence counselling or better still; if they are going to the field, it is not the counsellor-tester that will do the correct adherence! Let them go with adherence counsellor who would give, at least, the initial talk. It might not be that lengthy, but that person will now link to that person in the facility who will now give the full dose of the adherence counselling” (SERVICE PROVIDER_Lagos).

#### Follow-up and feedback

Service providers reported that creating a feedback mechanism to ensure that clients could share their experiences with medication use, side effects and coping strategies helped clients work through the most challenging periods after initiation of treatment. Some service providers highlighted the importance of empathy and follow-up calls in facilitating adherence. The follow-up calls served as opportunities to remind clients of their next appointments.

“For those who are enrolled…maybe at the first month; who are brought in newly, they normally have issues – side effects, which are the normal adverse drug reactions to the drugs but because every member of the clinic team, is an adherence counselor, there’s this feedback we get from there or we put calls across to them; the first day, the third day, the seventh day, the twelfth day and the fourteenth day just to find out how they’re doing on the drugs. So, because of this adherence call, they’re able to…they’re stable on ART**”** (SERVICE PROVIDER_Benue).

“A client is more relaxed when a counselor talking to him shows him his or her drugs and tell him your history and of course, they see you doing well, they have that confidence; and we also establish client-counselor relationship. I am the counselor. They are the client. I give them my telephone number, my name, I always ask them to feed me back – how they’re feeling especially when they are being…initiated on drugs in case of any side-effects and I’ll give them… I open my phone okay; I gave them the opportunity to call me at any time even in the midnight to talk to them. So, we have that relationship**”** (SERVICE PROVIDER_Lagos).

#### Client mobility and issues with tracking

Client mobility was a critical challenge for adherence because it made tracking difficult. FSW frequently changed their location due to issues relating to police raids of brothels, demolition of brothels or the need to change location for personal safety. These issues limited the tracking efforts of providers and further worsened by the regular change of phone numbers as a personal security measure.

“Clients defaulting from ART, in the part of the FSW, it’s a bit high because they change their location or sometimes, the place they are will be raided or broken down or there will be fire incident; and you see them scatter…” (SERVICE PROVIDER_Lagos).

“The only thing is their mobile, because they are not always in a physical location. They steal their phone a lot, and they don’t replace it, so contacting them sometimes is difficult for us and especially the FSW they stay in the brothel and you know how Lagos is, they go there and raid them, so sometimes it’s very difficult for us to make it. So, I think that’s just the major challenge” (SERVICE PROVIDER_Lagos).

Although some clients managed the issue of mobility by trying to access multiple health facilities for medication, it was difficult to keep track of their records. When clients reported that they were collecting medications from other facilities during the period when they did not attend their clinics, it was difficult to verify that they were adherent during the period they missed their appointment.

“So, recently we’ve been noticing adherence issues that we cannot monitor. You see a lot of clients coming around and maybe they miss their appointment, not necessarily their drug but their appointment. Adhering to drug and not adhering to appointment is not full adherence because you’re also meant to adhere to clinic visit and appointment… I think there should be a way to manage this. Not by clients having access to several facilities! So, you cannot tell who is telling the truth, who has missed medication or who has not missed; and with that, you cannot manage adherence issues. It has even led to so many other issues… maybe a client that has failed on first-line… and was placed on second-line; another facility might start the person on first-line, not knowing that this person has failed…on first-line” (SERVICE PROVIDER_Lagos).

#### Client’s sense of entitlement

Client’s sense of entitlement was reported as a challenge by service providers. Often, clients were unwilling to come for ART appointments unless there was assurance of reimbursement of transportation cost. In addition, clients made demands from service providers because they felt that the ART programs were donor funded and provided a wide range of benefits they could access when they visited the clinics.

“What I don’t like is not the program; it’s the clients themselves – you understand? There is this sense of entitlement that has come into the program. They feel… it’s their right, it’s their entitlement… because now we are funded… donors that fund us… ‘This is what I am entitled to so, don’t tell me you’re not going to do that’. So, that aspect pisses me off. I really don’t like when… clients feel that I’m entitled to this thing so if it is not up to what you’re giving me before, there’s a problem. You must give me this… you must give me 1,000 Naira… before I do anything with you” (SERVICE PROVIDER_LAG).

“There are times, we have to follow up clients – ‘Oh, come for your drugs’ and a client would ask you ‘If I come, will you give me my transport money back?’ So, to me it’s difficult when we get to your area; you don’t pick up our calls. So, it makes our jobs difficult because if only they understand that… we are trying to make life easier for them and key into that vision that we have…it’s not my entitlement, they’re helping me out’ it will make my job easier, my retention would be beautiful… But when you are having people that are difficult, you cannot reach that your 90%... You won’t get there. You are even lucky if you’re getting 60% retention. So, it’s actually a big challenge for me**”** (SERVICE PROVIDER_LAG).

## Discussion

This qualitative study explored clients and providers perception about facilitators and barriers to ART adherence among MSM and FSWs in Lagos and Benue states. The barriers identified by clients include low motivation to comply with medication regimen, work schedule, socioeconomic factors, stigma, negative provider attitude and distance to health facilities. Facilitators of adherence identified by clients include the desire to live a productive life, family support and support groups. Comprehensive adherence counselling and a strong follow-up/feedback system were factors identified by providers as key to facilitating adherence. These findings are consistent with findings from general population studies on barriers and facilitators of adherence [16, 18–20].

Although clients identified adherence counselling as key to developing a personal understanding of the importance of ART adherence, strategies to address constraints due to busy work schedule or frequent travel will strengthen their resolve to adhere to treatment. Addressing poor client motivation that is linked to forgetfulness, procrastination and missing of appointments may require an individualized approach that supports the integration of the treatment program into clients’ lives. A wide range of strategies from setting reminders, using calendars, peer and provider support programs have been documented in other studies [11, 14, 19, 20, 27]. Perceptions about the missed opportunities to earn income to meet basic needs due to time lost attending ART clinics as well as other economic considerations for transportation cost or feeding, may impact negatively on ART adherence as seen in this study. These findings are similar to those reported in other studies where poverty was a major reason for the inability to afford transportation cost and eat nutritious diet [27–33]. Programs that mitigate economic barriers improve appointment adherence [14, 28]. For example, a randomized control trial in Tanzania showed that clients who had received nutritional assessment and conditional cash transfer were more likely to be retained in care and adherent compared to those who received only nutritional assessment [34] In Malawi, unconditional cash transfer was associated with self-reported ART adherence [35]. In other studies in Uganda and Swaziland, ART clients who received rent support and incentives for clinical attendance respectively, had higher retention and lower mortality compared to those that did not receive these incentives [36, 37]. Transport assistance has also been shown to positively impact treatment outcomes, A study in India reported higher adherence rates verified by pill count, while another study in Zambia reported that the provision of free transport was associated with improved adherence [38, 39].

Similar to findings in other studies, family support was found to be invaluable in facilitating motivation and ease of compliance with ART treatment regimen [27, 30, 33, 40]. Other studies have also documented that lack of partner and family support could negatively influence ART adherence among people living with HIV/AIDS (PLWHAs) [9, 32]. In this study, clients expressed reservations about disclosing their HIV status to partners or family members who may not be supportive. While disclosure of HIV status may be beneficial in providing valuable support to clients to keep up with drug regimen and schedules, the process of disclosure should be individualized based on the client’s circumstances. Findings from other studies have shown that HIV disclosure could be a facilitator or barrier to ART adherence [4, 28, 41].

The value of support group meetings as a facilitator for ART adherence documented in this study reinforces the importance of peer driven strategies for implementation of KP HIV programs. Listening to peer success stories as well as discussing practical strategies to overcome challenges faced in ART treatment is an important motivating force [31, 42].

Real or perceived stigma could limit clients from accessing and adhering to ART if they are seen using the ART medication or visiting the clinics. Several studies have documented strategies clients adopt to avoid stigma such as enrolling in ART clinics that are far from their homes to avoid being identified by friends or service providers who are known to them [28, 29, 43–45]. This underscores the need to prioritise privacy and confidentiality as part of the treatment process. Stigma (real or perceived) could present as a barrier to disclosure and to ART adherence [27, 40, 41, 46].

Training health service providers on practical stigma reduction strategies, sexual orientation and gender identity, confidentiality and structuring health facilities to support privacy is key to facilitating adherence [4, 42]. Findings from this study show that the attitude of service providers was an important facilitator or barrier to ART adherence. Trust, communication and respect within the patient-provider relationship especially for KPs who face additional barriers accessing care than general population may be a critical influence factor for patient attendance at clinics and ART adherence. Friendly service provider attitude has been reported to facilitate the scheduling of ART appointments [9, 14, 30, 33, 47]. When clients feel disrespected by healthcare providers or their complaints are not adequately addressed, they may not be inclined to meet up with their appointments and this may hinder ART adherence [32–34]. Adequate, professional communication can help patients engage better with providers and receive clear information that will guide treatment planning.

Challenges with distance to health facilities and the prohibitive cost of transportation that resulted in participants skipping appointments may become barriers to adherence. The transport stipend participants received was a strong motivation to keep up with appointments and reflects innovative ways of facilitating adherence if the provision of stipends can be sustained. Community ART models that bring treatment closer to clients’ communities are also innovative approaches to addressing logistic barriers to uptake of ART. Unfortunately, some clients may not be inclined to medication pickup within the community due to issues relating to confidentiality and to avoid stigma associated with being seen by familiar faces.

Comprehensive adherence counselling when integrated into all service delivery points can be an effective way of improving client decision making about the care process. The challenges with providing comprehensive counselling at community level compared to facility level due to the pressure community service providers face can be addressed by providing counselling in phases. A hybrid of approaches can be considered to improve retention. The importance of feedback mechanisms constrained by issues with tracking of clients and client mobility can be addressed using peer to peer support groups. Community level interventions that support a stigma-free environment are critical to sustaining engagement in care.

### Study limitation

This study has some limitations. The study was based on client and service provider experiences about ART adherence and enrollment into care. Participants responded based on their perceptions about the care process. The study sites used in this research specifically provide services for KPs and consequently the views of clients and providers represented in this study may not represent the broader perspectives in another context. A small purposive sample of clients and providers was used and may lack broader generalizability. Findings from this research, however, provided critical insights for the policy and practice implications of setting up HIV treatment programs for KPs. Another limitation is reflexivity in which the researcher’s relationship with research participants with respect to views, inclinations, assumptions, and past experiences impact on the research process. Reflexivity ensures that the process of constructing knowledge takes into consideration, the interests and inclinations that influence how research is planned, implemented and interpreted [48]. The lead researchers ‘experience working with key populations for over a decade provided an understanding of the research context and approaches to working with participants. The researchers, however, were aware and acknowledged that their career experience, gender and position regarding the research may have influenced the research process. Recognising that these positions exist helped the researchers to consciously put them aside, document viewpoints and interpretations of issues during the entire research.

## Conclusion

The findings about the facilitators and barriers of ART adherence show that creating a supportive environment for KP HIV treatment is hinged on adequate empathetic engagement from providers and peers through comprehensive adherence counselling and support group meetings respectively. Innovative approaches to facilitating privacy and convenience of appointment schedules in a stigma free setting is key. Addressing challenges of distance to health facilities, the prohibitive cost of transportation and follow-up of clients requires sustainable strategies to ensure long term adherence and retention in care.

## Data Availability

Data is available upon request from the corresponding author.
